# Collective Behaviour in Video Viewing: A Thermodynamic Analysis of Gaze Position

**DOI:** 10.1371/journal.pone.0168995

**Published:** 2017-01-03

**Authors:** Kate Burleson-Lesser, Flaviano Morone, Paul DeGuzman, Lucas C. Parra, Hernán A. Makse

**Affiliations:** 1 Levich Institute and Physics Department, City College of New York, New York, NY, United States of America; 2 Neuromatters LLC, New York, NY, United States of America; 3 Biomedical Engineering Department, City College of New York, New York, NY, United States of America; Ghent University, BELGIUM

## Abstract

Videos and commercials produced for large audiences can elicit mixed opinions. We wondered whether this diversity is also reflected in the way individuals watch the videos. To answer this question, we presented 65 commercials with high production value to 25 individuals while recording their eye movements, and asked them to provide preference ratings for each video. We find that gaze positions for the most popular videos are highly correlated. To explain the correlations of eye movements, we model them as “interactions” between individuals. A thermodynamic analysis of these interactions shows that they approach a “critical” point such that any stronger interaction would put all viewers into lock-step and any weaker interaction would fully randomise patterns. At this critical point, groups with similar collective behaviour in viewing patterns emerge while maintaining diversity between groups. Our results suggest that popularity of videos is already evident in the way we look at them, and that we maintain diversity in viewing behaviour even as distinct patterns of groups emerge. Our results can be used to predict popularity of videos and commercials at the population level from the collective behaviour of the eye movements of a few viewers.

## Introduction

It is often said that our preferences and biases influence the way we see the world. This is literally true when viewing static images, where prior preferences influence our gaze, and inversely, our point of gaze can affect our subsequent judgments of what we see [1, 2]. Thus, it is conceivable that our differing preexisting preferences are only reinforced by a biased view of the world, even during such simple behaviours as looking at images. On the other hand, viewers seem to have a remarkably similar way of looking at dynamic visual stimuli. Previous studies have used eye-tracking to explore connexions between visual stimuli and viewer attention [3–5]. Well-produced movies, in particular, effectively synchronize eye-movement trajectories across viewers [6, 7]. Perhaps more importantly, movies elicit quite similar brain responses across viewers [8], especially when the audience is engaged and attentive [9, 10]. Interestingly, this similarity of brain responses can predict various preferences of large audiences [11].

Perhaps then, well-produced video material also synchronises judgments, and so we asked whether similarity of eye movements is predictive of collective preference ratings. To answer this question, we selected videos with high production value that have been viewed by large audiences. We used commercials aired during the 2014 Super Bowl championship game of the National Football League (American football), watched by over one hundred million people [12] and for which population-level preferences ratings are readily available.

The quantitative analysis of these data is based on *homophily* and *inter-subject correlation* (ISC). In sociology, the study of homophily has gained considerable attention by showing that people relate best to those who they perceive as similar to themselves, those who display similar thinking [13–15] or even similar body language [16]. We will analyse homophily of preference, measured as the similarity in subjective ratings of commercials. We find that the most popular videos also have similar preference ratings.

In neuroscience, the study of inter-subject correlation has demonstrated that successful inter-personal communication and similar points of view are accompanied by correlated brain activity between individuals [17, 18], and that emotional and memorable stimuli elicit higher brain synchrony between individuals [17, 19]. Here, we analyse inter-subject correlation of eye movements. To gain a deeper understanding of these correlations, we will employ tools from statistical mechanics, which aim to explain emergent properties out of the local interactions between elements (individual people in this case). These modelling tools have been applied to biological systems demonstrating fundamental phenomena such as the emergence of collective behaviours and criticality [20–23]. We will demonstrate that some videos place the audience at a critical balance between perfect alignment and randomness, at which point distinct groups of viewing behaviours emerge. Those videos close to criticality exhibit strong communities of viewers (high modularity in the network of interactions). Our results indicate that is possible to predict population-level popularity of commercials and videos from the thermodynamic analysis of the collective behaviour of eye movements of a few viewers.

## Results

### Experimental design and data acquisition

**Fig 1:**
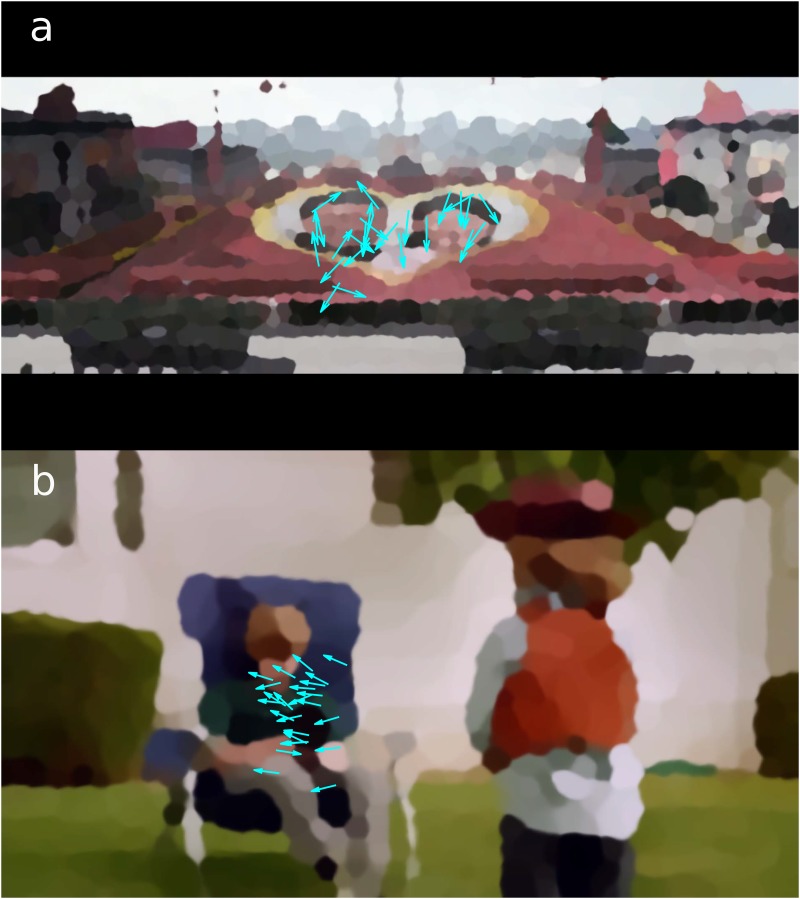
Subjects’ gaze positions and movement directions. (**A**) Vectors representing the subjects’ gaze positions superimposed upon a map of shapes and colours in 19th second of “Make Love, Not War”, the Axe Body Spray commercial. (**B**) Vectors representing the subjects’ gaze positions superimposed upon a map of shapes and colours in 4th second of “Cowboy Kid”, the Doritos commercial. In (**A**), viewers are less focussed and eye movement directions are less coherent. This video had a lower Ad Meter rating (4.92). In (**B**), several distinct groups of viewers can be seen, each group gazing in a different direction from the others. This video had a higher Ad Meter rating (7.58) and was rated highly in a number of other national polls related to the 2014 Super Bowl commercials [12, 24–26].

Twenty-five (25) study participants watched 65 commercials, which were broadcast on television during the 2014 Super Bowl, arguably the most popular sporting event in the United States. Each participant watched the videos individually while their vertical and horizontal eye gaze position was recorded. The subjects’ gaze positions during one instant each from a pair of videos, superimposed upon a map of relevant shapes and colours in order to provide a point of reference for where the subjects were looking, are shown in Fig 1. A movie of the gaze trajectories of all participants during the course of the video in Fig 1B can be seen in S1 video. Videos were presented to each subject in random order. At the end of each video, participants were asked to rate how much they liked it on a scale of 0 to 10. For 56 of these videos, population-level ratings were available through an online survey of thousands of viewers, USA Today’s Ad Meter, which has become a standard metric in the advertising industry. Details of the experimental procedures are presented in Methods.

### Viewer opinion of videos is predicted by eye movements

**Fig 2:**
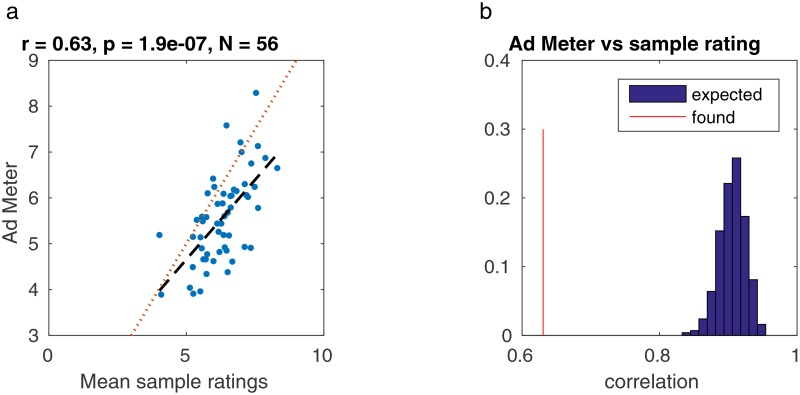
Comparison of Ad Meter ratings with ratings from our test group. (**A**) The Ad Meter ratings are systematically higher than the ratings from our sample of test subjects. Additionally, correlation between Ad Meter and sample ratings is *r* = 0.63, which is significantly lower than expected from a homogeneous sample of *N* = 25 raters. (**B**) To establish this, we drew at random 25 ratings with mean taken from the population and added Gaussian noise with standard deviation taken from the sample group. On average we obtained *r* = 0.91. Drawing repeatedly in this manner 10^5^ times, we did not find a single case with correlation smaller than this value. Thus we can dismiss the hypothesis of a homogeneous sample with *p* < 10^−5^).

First, we use Pearson correlation to compare the ratings of our subjects to the larger-scale population data available from the Ad Meter (Fig 2, *r* = 0.63, *p* = 1.9 × 10^−7^, *N* = 56). There is a lower-than-expected correlation between population and sample ratings which is perhaps due to the experimental sample size being 0.4 percent of the size of the Ad Meter panel. Previous work in neuroscience regarding inter-subject correlation of video viewers has proposed that in such a small group of people, individual preferences—essentially “noise” —will have a much greater effect on the group’s average opinion, whereas in a much larger group of people, the “noise” will reduce because competing individual preferences will cancel each other out [11].

**Fig 3:**
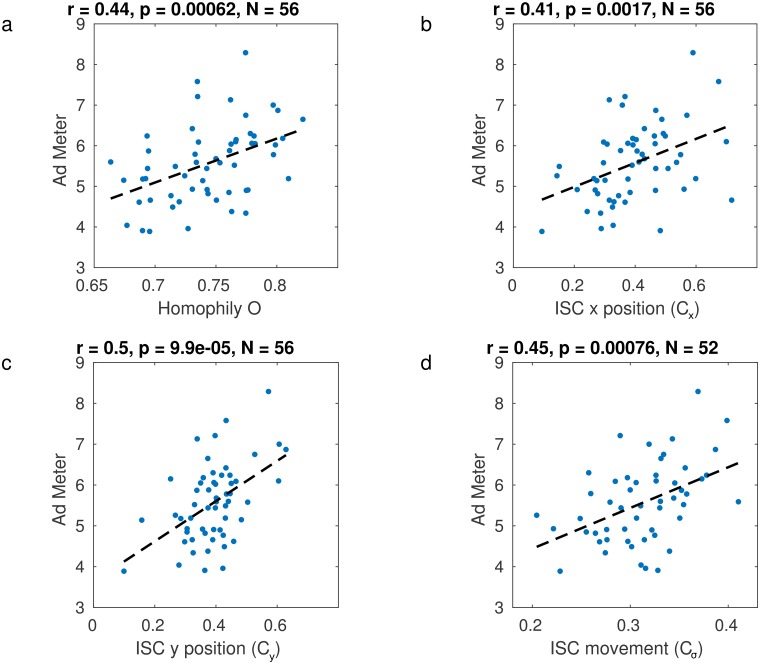
Ratings and correlation of eye gaze position. (**A**) Homophily in ratings correlates with population-level preferences. Each point indicates a video commercial from the 2014 Super Bowl. Homophily is measured as the similarity in ratings between the 25 study participants. Homophily of 1 indicates perfect agreement among all viewers in the ratings of the video. Population preferences are given with the Ad Meter ratings, which were available for 56 of the commercials. (**B**) and (**C**) indicate that population-level preference are predicted by similarity of eye gaze positions (inter-subject correlation *C*_*x*_ and *C*_*y*_ of horizontal and vertical eye gaze positions). (**D**) Similarity of eye movement direction across viewers (*C*_*σ*_) correlates with Ad Meter ratings. For example, the video shown in Fig 1B had among the highest *C*_*σ*_ of all videos and received high popularity ratings (Ad Meter = 7.58).

We then compute homophily of ratings based on the numerical difference of the ratings between viewers (see Eq (2) in Methods) and compare this to the Ad Meter ratings at the large-population level (Fig 3A). There is a significant Pearson correlation (*r* = 0.44, *p* = 0.00062, *N* = 56) indicating that the most popular videos also had a more uniform appeal in our sample. Previous literature suggests that popular commercials elicit correlated brain responses in viewers as measured by EEG [11]. To determine if this is already apparent in a similar point of gaze across viewers, we quantified the correlation of vertical and horizontal eye positions across viewers (Pearson correlation coefficients of *x*- and *y*-eye position coordinates, ISC, averaged over all viewer pairs, Eq (3) in Methods). This similarity of eye position indeed correlates with Ad Meter ratings (Fig 3B and 3C).

Instead of gaze position, we focus next on eye movement direction of viewer *i* ${\vec{\sigma }}_{i}\left(t\right)$ (Eq (4) in Methods). Direction of eye movement is important for two reasons. First, the literature on attention emphasises reorienting of attention to new locations, i.e., saccades rather than content of fixations. Secondly, the variable ${\vec{\sigma }}_{i}\left(t\right)$ allows us to use models from statistical mechanics that have been developed in the context of movement of independent agents (flocks of birds [20, 21] that are not unlike our current situation with “flocks” of viewers). These models capture the pairwise correlations *C*_*ij*_ between directions of viewers *i* and *j* ${\vec{\sigma }}_{i}\left(t\right)$ and ${\vec{\sigma }}_{j}\left(t\right)$ (Eq (5) in Methods). These pairwise correlations can be averaged over all pairs of viewers to capture the overall similarity of eye movements in the group as *C*_*σ*_ (Eq (7)). We find again a solid link between similarity of eye movements, *C*_*σ*_, and population ratings (Fig 3D, *r* = 0.45, *p* = 7.6 × 10^−4^, *N* = 52).

### A statistical model to explain viewing behaviour

To gain deeper insights into the collective behaviour of viewers, we build a statistical model for the “interactions” of eye movements of the group, in order to uncover any dependencies between variables (here, the eye movements of two different viewers) that may not be immediately apparent from the inter-subject correlations. Furthermore, this model would disregard any inter-subject correlations between two subjects which only arise as a result of each having some similarities with the viewing patterns of a third subject (a more in-depth explanation is in the Discussion section). Arguably, the minimal model is one where the distribution of directions is entirely random except that it reproduces the observed mean direction ${\vec{\mu }}_{i}$ and correlations *C*_*ij*_. This is known as a Maximum Entropy model [27], which in this case has the following probability distribution [22]:

$$\begin{array}{c} p\left(\vec{\sigma }\right)\propto \exp\left(-\sum_{i}\;{\vec{h}}_{i}\cdot {\vec{\sigma }}_{i}-\sum_{i,j>i}\;J_{ij}{\vec{\sigma }}_{i}\cdot {\vec{\sigma }}_{j}\right). \end{array}$$ (1)

**Fig 4:**
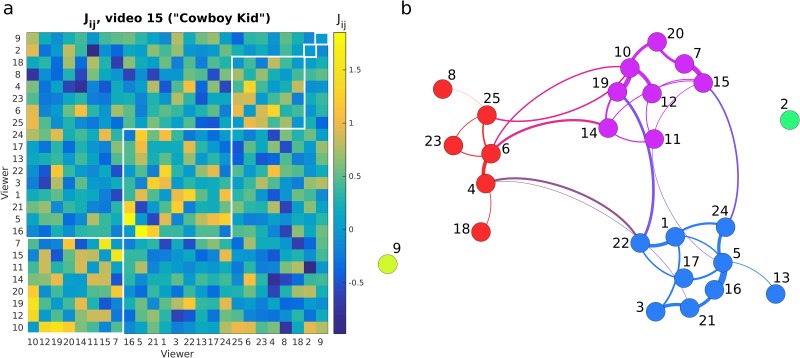
Interaction strength *J*_*ij*_ between viewers and corresponding network structure. *J*_*ij*_ captures similarity of the eye movement patterns that are uniquely shared between viewers *i* and *j*. Here, we show the interactions and network for Video 15, the “Cowboy Kid” commercial by Doritos. In (**A**) participants have been arranged in groups with similar interactions, corresponding to the groups shown in (**B**). Groups are outlined in white. The participant numbers are listed along the vertical axis. Self-interactions *J*_*ii*_ are set to zero. The interactions shown here have high modularity and correspond to the video shown in Fig 1B. Each node in the network structure corresponds to one viewer, and thickness of lines connecting nodes indicates interaction strength *J*_*ij*_. Weak interactions have been omitted for clarity.

**Fig 5:**
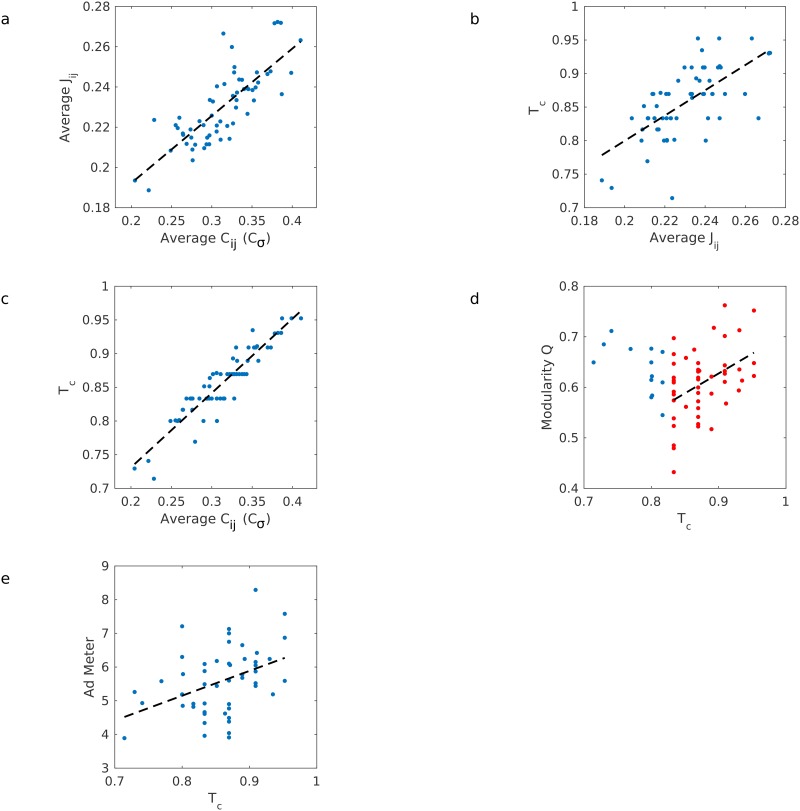
Critical temperature. (**A**) Correlations *C*_*ij*_ are used to estimate interactions *J*_*ij*_. Here average values across all pairs of viewers are shown. (**B**) Interaction strengths are used to compute critical temperature. (**C**) There is a tight relationship between *T*_*c*_ and average correlations (*r* = 0.86, *p* = 4.83 × 10^−27^, *N* = 61). (**D**) The videos closest to criticality (*T*_*c*_ > 0.82, plotted with red dots) show a correlation between critical temperature and modularity (*r* = 0.43, *p* = 0.0021, *N* = 48); at higher temperatures, more distinct and cohesive communities emerge. (**E**) Ad Meter correlates with critical temperature (*r* = 0.41, *p* = 0.0028, *N* = 52); when there is large-scale agreement in viewing behaviour, a video stimulus will tend to be rated more highly.

For each video we fit model parameters ${\vec{h}}_{i}$ and *J*_*ij*_ so that the means and correlations of this joint distribution equal the observed means ${\vec{\mu }}_{i}$ and correlations *C*_*ij*_ (see “Maximum Entropy Method” in Methods, and S1 Appendix). Since there is no net drift of gaze while the subjects watch the commercial, the parameter ${\vec{h}}_{i}$ is always very close to zero. Fig 4 shows the resulting *J*_*ij*_ for one of the most popular videos of the set. We will refer to these parameters *J*_*ij*_ as the “interactions” between viewers and will justify this term in more detail in the discussion. For now, it suffices to say that we think of it as a measure of similarity of the eye movement patterns that are uniquely shared between viewer pairs. This stands in contrast to *C*_*ij*_, which captures all correlations that may be shared across many viewers. Since we are interested in whether there are groups of viewing behaviours, we analyse the network structure of these interactions *J*_*ij*_. We use a conventional clustering technique [28] to group together subjects with similar entries in the interaction matrix *J*_*ij*_, as shown in Fig 4. Separate communities can be seen in the groups of blocks along the diagonal; each group is a different community, and these are the same groups seen in the snapshot in Fig 1B. For these networks corresponding to each video, we can compute modularity *Q* (Eq (10) in Methods) and we find that all videos have relatively high modularity (Fig 5D). High modularity means that there are groups of viewers with patterns of eye movement directions unique to each group but common among group members. Lower modularity means that the groups of viewers are less distinct. The corresponding network of viewers in Fig 4B, defined by the strength of *J*_*ij*_, shows the formation of communities in Fig 1B.

The tools of statistical mechanics allow now for the analysis of the subjects’ emerging viewing behaviour. We ask, what would have happened if viewers had been more or less coupled than what we actually observed? Since we have an explicit model for the statistics of movement direction, Eq (1), we can readily simulate this by scaling interaction strength *J*_*ij*_ and measuring the resulting correlations *C*_*ij*_. Classical statistical mechanics argues that when interactions *J*_*ij*_ are very strong, the behaviour of the coupled elements should fall in perfect lockstep, while the remaining fluctuations (as in vibrations around a “crystal”) are not correlated between elements. On the other hand, if interactions are very weak, then behaviour is entirely random and correlation approaches zero as in a “fluid”. Thus, correlation is zero at both extremes, but in some intermediate scale, correlation is maximal. In statistical mechanics, this scale is referred to as “critical temperature” *T*_*c*_ (higher temperature than *T*_*c*_, or equivalently low interaction strength, corresponds to a very disordered state, whereas lower temperature than *T*_*c*_, equivalent to strong interaction, corresponds to a very ordered or “crystal”-like state). Critical temperature is estimated here by scaling *J*_*ij*_ for each video and determining a point of maximal inter-subject correlation marking the emergence of collective behaviour (see Methods for more information).

Fig 5 shows the resulting critical temperatures *T*_*c*_ of all videos and their relationship to various order parameters. In this figure, the operational temperature of viewing is set to 1, thus *T*_*c*_ equal to 1 implies “critical” viewing and *T*_*c*_ less than 1 implies a “fluid” state. There are two interesting results in these figures. First, there is a very tight link between average correlation and critical temperature (Fig 5C). This is not an obvious result when considering that temperature is computed from *J*_*ij*_, which in turn is computed from *C*_*ij*_, and each step in this process does not exhibit such a tight link. The second interesting result is that all *J*_*ij*_ with high critical temperature tend to also have high modularity. Note that *T*_*c*_ = 1 means that *J*_*ij*_ of the video are at the critical point without requiring any scaling. What this tells us is that high-*T*_*c*_ videos close to 1 are close to a critical point, where any stronger interaction would make all viewers behave the same (crystal-like) and any less would make their behaviour entirely random (fluid-like). For the videos near this critical point, groups emerge with similar collective viewing patterns among the group but distinct from those in other groups, i.e., they have high modularity (Fig 5D). For videos with high *Tc* (*Tc* > 0.82) there is a clear correlation of critical temperature with modularity (*r* = 0.43, *p* = 0.0021, *N* = 48, red points in Fig 5D). That is, these videos elicit from their audience cohesive responses within different groups, and diverse viewing behaviours between the groups. The closer to criticality, the greater the modularity and the popularity of the videos due to the emerging collective behaviour of the interactions, and it is interesting that it is precisely these videos that tend to have higher agreement in attention reorienting (*C*_*σ*_). As is shown in Fig 5E, they are also the most popular videos on the broader scale, as videos with a higher critical temperature tend also to have higher Ad Meter ratings (*r* = 0.41, *p* = 0.0028, *N* = 52). It is important to note in Fig 5D that we discount the videos with a lower critical temperature, which are also those videos with lower average couplings *J*_*avg*_ (as in Fig 5B). Since we find the modularity of each network’s adjacency matrix at a threshold *J*_*o*_ where a giant connected component emerges (see Methods section, “Modularity”), it is possible that the calculation of modularity was affected by the lower threshold value that accompanies lower overall couplings. That is, these lower-*J*_*avg*_ networks tend to have one large but very weakly-connected giant component and some unconnected nodes, rather than several distinct and strongly-connected groups as in the higher-*T*_*c*_ videos. The adjacency matrices are binary (a value of 1 denotes any link, no matter how weak) and simply show a large giant component and some single nodes without taking into account the weakness of connexions, which may have skewed the calculation of modularity for lower-*T*_*c*_, lower-*J*_*avg*_ videos. However, we expect that modularity should overall decrease with decreasing critical temperature *T*_*c*_, which we test by shuffling the gaze trajectories to randomise them and destroy correlations between subjects. When we do analysis on the randomised videos, we indeed find *T*_*c*_ going to 0 and zero modularity (see S4 Appendix for more details).

## Discussion

The goal of this work was to determine if preference of an audience, including overall popularity and diversity of opinions, can be inferred already from the way they look at videos. We find that the most popular videos elicited highly correlated eye gaze positions. Videos were close to a critical level of correlation where distinct groups of viewing patterns start emerging without bringing all viewers into lock-step. Interestingly, similarity of eye movements also predicted similarity of opinions (homophily). In total, our results suggest that diversity of opinion is already evident in the way we look at the world, and that we maintain diversity even as distinct viewing patterns emerge. Our main results follow.

### Popularity and inter-subject correlation

We have used commercials aired during the Super Bowl as they provide a natural experiment with some level of homogeneity: There is a large number of similar videos produced for and aired in the same context, with preference ratings uniformly collected from a large number of viewers. These Ad Meter ratings have often been used as a benchmark for marketing research, and various predictors of Ad Meter ratings have been proposed, although rigorous evaluations are rare. A recent study demonstrated that similarity of neural responses to video is predictive of popularity in large audience including Ad Meter ratings, Nielsen ratings and even tweeting frequency [11]. The measure used there is inter-subject correlation of electroencephalographic responses. There is rich literature on such inter-subject correlation of brain signals in response to videos [8, 9, 17–19]. One novel aspect of the present study is that we use ISC of eye movements instead. We analyse both ISC of gaze position, which is predictive of Ad Meter ratings, as well as ISC of movement direction, which emphasises reorienting of attention in the dynamic stimulus. Importantly, the present study is the first to emphasise diversity and homophily of opinions, and how these relate to the way we look at videos and their concomitant popularity.

### Statistical mechanics model of correlated behaviour

When analysing eye movement direction, groups of viewers emerge who share unique patterns of reorienting behaviour. This group behaviour is not readily observed in the raw correlations between subjects, only becoming apparent when we model the correlated behaviour as the result of a system of coupled viewers. To our knowledge, this is an entirely novel way of analysing similarities in human behaviour, and is an important theoretical contribution of this work. We build on previous statistical mechanics approaches to other biological systems: similar analysis has also been used to describe flight velocity in a flock of birds [20, 21] whose movements are not unlike those of the “flock” of eyes shown in Fig 1 and S1 video. In flocks of birds, flight direction appears to be dominated by nearest-neighbour interactions, which lead to remarkably coordinated collective behaviour of the flock. In particular, interactions are close to a critical point, where flight direction correlates far beyond the nearest neighbour, and where the collective behaviour of the flock is particularly sensitive so that it can respond quickly to disturbances [20, 22]. We find that the interaction of viewers for the most popular videos is at a similar critical point. Viewers are poised to respond immediately and cohesively to a change in the stimulus. This implies that they are attentive and focused on the video, which is consistent with recent findings that ISC in rapid brain responses is strongly modulated by attention [10]. Thus, we argue that correlated behaviour, just like correlated brain responses, is indicative of the attentional state of the audience.

### Interpretation of “interaction” between viewers

We have referred to model parameters *J*_*ij*_ as “interactions” between viewers and analysed the corresponding network of interactions. But what exactly is meant by “interaction”? Strictly speaking *J*_*ij*_ are parameters of the probability distribution given by Eq (1). In statistical mechanics a probability distribution of the form Eq (1) naturally arises for interacting particles, and parameters *J*_*ij*_ express the strength of interactions between pairs. It has been shown in previous papers that a thermodynamic model is applicable to different biological systems, such as neurons and flocks of birds [20–23], where collective behaviour can be seen among independent agents that interact as a group. Since collective behaviour among subjects’ gaze trajectories was observed when they watched some of the videos, we followed these prior studies and applied a thermodynamic model found using Maximum Entropy methods in order to determine if the videos during which the subjects behaved more collectively had any underlying properties which would differentiate them from the other videos. One such underlying property is long-range interactions within the group that are indicative of a critical point, as in the example of a flock of birds moving as a cohesive group with trajectories correlating beyond nearest-neighbour interactions [20, 21]. Another is the nature of “true” interactions between subjects, which can only be uncovered using a thermodynamic model. Two subjects, for example, may appear to have a somewhat strong correlation *C*_*ij*_, which is in reality only the result of each subject’s gaze trajectory being similar in some way to that of a third subject rather than to each other.

Since the subjects do not physically interact with each other during the viewing, one may think of the video as a medium through which ${\vec{\sigma }}_{i}$ couples with ${\vec{\sigma }}_{j}$ with strength *J*_*ij*_. In probability theory the same distribution arises for variables that are correlated, and *J*_*ij*_ captures their conditional dependence. One way to think about these dependencies is to imagine our test subjects as representatives of a prototypical viewing pattern. To further expand upon the hypothetical situation mentioned above, imagine viewer A and viewer B both sharing the viewing pattern represented by prototypical viewer C, for some fraction of the time. In the absence of this period of time, perhaps viewers A and B have nothing in common, and thus no longer correlate. While correlations *C*_*ij*_ are a promiscuous measure capturing correlations between all pairs of viewers, *J*_*ij*_ would in this case only have interactions between A and C and between B and C, but not between A and B, who do not share anything in common beyond what they share with C. In probability theory we would call A and B conditionally dependent on C, but conditionally independent from each other. For physical systems of interacting elements it would mean that A and B do not interact directly, but correlate only because they interact with C. Thus, the observed correlations can arise via a chain of interacting pairs without needing direct interaction between all. In that sense, global behaviour can arise from many interacting pairs and without global orchestration. In the present case, interaction strength between prototypical viewing patterns captures the fraction that two viewing patterns uniquely share, and the global correlations arise from a combination of such unique viewing patterns.

### Homophily in eye movement and preferences

The first finding with regards to homophily (inter-rater agreement in our sample group) is that it correlates with popularity ratings (Ad Meter ratings). This trend is somewhat to be expected since the more highly-rated videos need to have almost uniformly high ratings to score well on average, whereas the videos that were not rated as highly had a mix of ratings. As is often the case, more “acceptable” stimuli are uniformly liked, while those that may be in some way controversial are more polarising, inspiring a wider range of responses. We find that ISC of reorienting behaviour is a predictor of the popularity of the video. More importantly, it is also a predictor of homophily of opinions. If people view a video in similar ways, they are more likely to have similar, and positive, responses to the video. As such, these results do extend previous findings that similarities in physical behaviour among humans is correlated with better rapport, leading to tighter communities [16].

### Modularity, popularity and criticality

**Fig 6:**
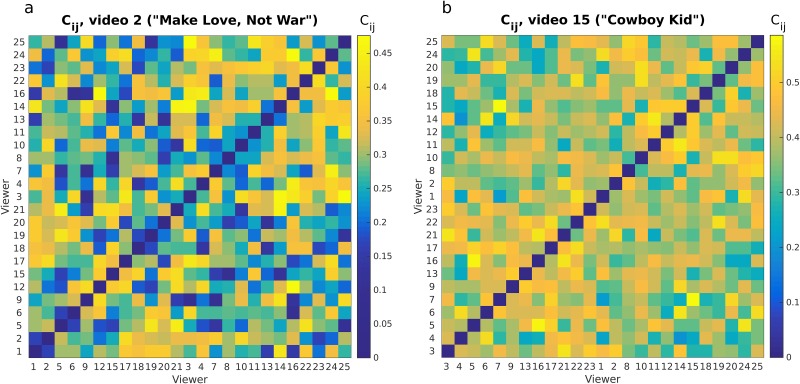
Pairwise correlations of eye movement directions *C*_*ij*_. Correlations are shown for the videos in Fig 1; (**A**) corresponds to the video in Fig 1A (the Axe commercial “Make Love, Not War”) and likewise for (**B**) (the Doritos commercial “Cowboy Kid”). Each row and column indicates a viewer, and viewers have been sorted to group rows/columns with similar entries together using a conventional clustering algorithm [28]. Self-correlations are set to zero for clarity.

An important observation of this work is that similarities in attentional orienting emerge between viewers while diversity between groups is preserved. This is formalised with the measure of modularity, which captures the strength of communities within a network [29, 30]. Here, the modularity is found from the interactions *J*_*ij*_ rather than from pairwise correlations of gaze direction. It is not surprising that strong average *C*_*ij*_ leads to clear modular networks in interactions *J*_*ij*_, as those videos with strong average correlations also displayed strong average interactions. However, the deeper community structure [28, 31] found in the interactions is not immediately apparent in pairwise correlations of the gaze direction (see Fig 6). This is likely due to the presence of “noise” in the raw data such as an apparent correlation *C*_*ij*_ between two subjects which only arise from their each being similar to a third subject; these two subjects would not have a strong interaction *J*_*ij*_, leading to differences in the community structure of *C*_*ij*_ and *J*_*ij*_ for the same video. Higher modularity means that the viewing patterns within a group are similar yet diverse across all groups. Using tools from statistical mechanics we identify a critical scale of interaction strength characterised by the critical temperature *T*_*c*_. An important feature of the high-*T*_*c*_ videos is the emergence of distinct communities among the network of viewers. This means that, rather than having the same collective behaviour across all 25 subjects for a given video, close to criticality there are instead several separate communities among viewers, each community behaving collectively within itself while interacting with the video in a different way from the other communities. The emergence of communities around the critical point coincides with the increase of the popularity of the video at the broader population level and suggests that the engagement of videos is already elicited in the collective behavior evident in the gaze interactions of viewers. In turn, the distance to the critical point obtained from the thermodynamical modelling of the eye-movement directions can be used as a predictor of popularity when the video is viewed by larger audiences.

## Methods

### Experimental methods

#### Subjects and stimuli

Twenty-five (25) subjects across a range of ethnic, gender, and age demographics (see S2 Table for details) were instructed to watch a set of 65 commercials that had aired during the 2014 Super Bowl. Procedures were approved by the Western Institutional Review Board (Puyallup, WA). Prior to the start of the experiments, all subjects gave written informed consent. Videos were shown in random order. At the end of each video participants were asked “On a scale from 0-10, rate how much you liked the commercial. 0 is strongly disliked, 10 is liked very much”. A continuous visual analogue scale was presented as a slider ranging from 0 to 10. Video commercials were of variable duration (of the 65 videos 3 were 10s long, 35 were 30s, 1 was 45s, 23 were 60s, 2 were 90s, and 1 was 120s). Gaze position data were taken during the commercials at a rate of 250 measurements per second (right eye only using EyeLink 2000, SR Research). The screen dimensions were 1440 by 2560 pixels and gaze position was given in terms of pixels.

#### Preprocessing for gaze positions

Data was preprocessed using MATLAB. The gaze data initially contained some eye-movement artifacts that resulted from partial occlusion of the pupil, and missing data due to brief lapses in data collection. We low-pass filtered the eye position data with a zero-phase 80 millisecond triangular window, which implicitly also marked data 40 milliseconds before and after as missing data. Participants which had more than 20% of missing data in a given video were excluded from further analysis for that video. All samples with missing data were set to 0. After this, a sparse principal component analysis [32] was run on each coordinate and video separately combining data from all available subjects. This step effectively inserted a linear interpolation from the over viewers for outliers and missing samples. The three videos of length 10s were omitted due to excessive noise. Those videos longer than 30s were broken down into 30s increments, and preprocessing and analysis were run on each increment separately. Afterwards, quantities such as couplings *J*_*ij*_ and critical temperature *T*_*c*_ were averaged over the increments of the long videos.

#### Ad Meter

In addition to the subjects’ individual ratings, we also used publicly accessible data on general opinion of the video via a metric called “Ad Meter”, in which people viewing the Super Bowl in real time rate the commercials online on a scale from 0 to 10. The Ad Meter is run annually by USA Today during the Super Bowl; panelists from a wide range of demographic groups volunteer to give their ratings of each advertisement as they air [24]. In 2014 there were approximately 6200 panelists [12]. Mean ratings in our test group correlate with the ratings of the Ad Meter population (*r* = 0.63, *p* = 2 * 10^−7^, *N* = 56). However, correlation is significantly lower than expected for a sample of 25 raters, indicating that the population and our test sample are not homogeneous (see Fig 2). Of the 65 commercials, only 56 have Ad Meter ratings, and the eye movement data was of sufficient duration to measure correlation of eye direction (30 seconds or more) for 62 videos. The intersection of the two gives 53 videos. The various analyses always include the maximum set of videos possible within these limitations.

### Homophily of opinion about a video

The homophily, or degree to which pairs of participants think alike about the video, is calculated as follows [14]:

$$\begin{array}{c} O=\frac{1}{N(N-1)}\sum_{i=1}^{N}\sum_{j\neq i}1-\frac{|p_{i}-p_{j}|}{p_{max}} \end{array}$$ (2)

with *p*_*i*_ ∈ [0, *p*_*max*_] representing the preference rating of participant *i* on a given video. 0 represents the least favourable opinion, and *p*_*max*_ represents the most favourable opinion of the video. This definition of homophily has a maximum value of 1 when all viewers exactly agree and has a minimum value of 0.5 when half the subjects rate 10 and the other half rate 0 (maximum disagreement in the group).

### Pairwise correlation of gaze position and velocities

The first analysis in Fig 1 focusses on horizontal and vertical gaze positions on the screen, denoted *x*_*i*_(*t*) and *y*_*i*_(*t*) for the *i*th viewer. To capture similarity in point-of-gaze we measure Pearson correlation coefficients between pairs of viewers and average across all pairs. Here, for horizontal eye position:

$$\begin{array}{c} C_{x}=\frac{1}{N(N-1)}\sum_{i}\sum_{j\neq i}\frac{{\left\langle \left(x_{i}\left(t\right)-{\bar{x}}_{i}\right)\left(x_{j}\left(t\right)-{\bar{x}}_{j}\right)\right\rangle }_{t}}{\sqrt{{\left\langle {\left(x_{i}\left(t\right)-{\bar{x}}_{i}\right)}^{2}\right\rangle }_{t}{\left\langle {\left(x_{j}\left(t\right)-{\bar{x}}_{j}\right)}^{2}\right\rangle }_{t}}}\;, \end{array}$$ (3)

where ${\langle f\left(t\right)\rangle }_{t}=1/T\sum_{t=1}^{T}f\left(t\right)$ is the conventional time average (excluding samples marked as missing data), and mean value is denoted as ${\bar{x}}_{i}={\langle x_{i}\left(t\right)\rangle }_{t}$. A similar expression is obtained for vertical eye positions *C*_*y*_.

Motivated by previous research on correlations of velocity of independent agents (flocks of Starlings) [20, 21] we compute the eye movement direction ${\vec{\sigma }}_{i}\left(t\right)$ from its 2-D velocity ${\vec{v}}_{i}\left(t\right)=0.5\left({\vec{r}}_{i}\left(t+1\right)-{\vec{r}}_{i}\left(t-1\right)\right)$:

$$\begin{array}{c} {\vec{\sigma }}_{i}\left(t\right)={\vec{v}}_{i}\left(t\right)/|{\vec{v}}_{i}\left(t\right)|\;. \end{array}$$ (4)

We also compute velocity for theoretical reasons. First, the correlation function (Eq (5)) must tend toward zero for very dissimilar values of velocity —two subjects’ gazes moving in completely different directions —and this is not satisfied by correlating only the gaze positions. Second, when we model the system, the Hamiltonian (Eq (8)) must be translationally invariant and have a lower bound so that the ground state is well-defined; this, also, is only satisfied for the gaze velocities.

For each video we compute the correlation of eye movement directions of two viewers *i* and *j*:

$$\begin{array}{lll} C_{ij} & = & {\langle {\vec{\sigma }}_{i}\left(t\right)\cdot {\vec{\sigma }}_{j}\left(t\right)\rangle }_{t}-{\vec{\mu }}_{i}\cdot {\vec{\mu }}_{j}, \end{array}$$ (5)

$$\begin{array}{l} \begin{array}{lll} {\vec{\mu }}_{i} & = & {\langle {\vec{\sigma }}_{i}\left(t\right)\rangle }_{t}. \end{array} \end{array}$$ (6)

Fig 6 shows *C*_*ij*_ for the two videos in Fig 1 with higher and lower modularity *Q* (defined below).

From this we compute again the average across all pairs:

$$\begin{array}{c} C_{\sigma }=\frac{1}{N(N-1)}\sum_{i=1}^{N}\sum_{j\neq i}C_{ij} \end{array}$$ (7)

This again represents an inter-subject correlation, in this case of eye-movement directions. It should be noted that this quantity in statistical mechanics is referred to as susceptibility and is an important order parameter of the system. It characterises how susceptible is the system of interacting particles to small external perturbations.

### Statistical mechanics of 2D directions in eye-movement

In statistical mechanics, a system of interacting units that are characterised as 2-D unit vectors —such as the eye movement direction vectors *σ*_*i*_ —is known as the XY model [33]. In the XY model the strength of their interaction is characterised by a scalar matrix *J*_*ij*_ and external perturbations by the “field” ${\vec{h}}_{i}$ acting on each unit *i*. The energy of such a system is given by

$$\begin{array}{c} E=-\sum_{i=1}^{N}{\vec{h}}_{i}\cdot {\vec{\sigma }}_{i}-\sum_{i<j}J_{ij}{\vec{\sigma }}_{i}\cdot {\vec{\sigma }}_{j}, \end{array}$$ (8)

In principle, all viewers “interact” with all other viewers through the viewing of the video quantified by *J*_*ij*_. Thus, the model corresponds to a fully-connected XY model [33]. In this model self-interactions *J*_*ii*_ are set equal to zero. At the microscopic level time-varying *σ*_*i*_(*t*) will affect other units *σ*_*j*_(*t*) such that the system is in perpetual fluctuation. On a long time scale the units will take on a range of values distributed according to Boltzmann distribution:

$$\begin{array}{c} P_{T}\left(\vec{\sigma }\right)\propto \exp\left(-E\left(\vec{\sigma }\right)/T\right)\;, \end{array}$$ (9)

where *T* represents the level of fluctuations in the system (high temperature implies large fluctuations, and at zero temperature fluctuations disappear and all units are “frozen”—unchanging). Note that the minimum probabilistic model necessary to explain correlations *C*_*ij*_ and means ${\vec{\mu }}_{i}$, the maximum entropy distribution (Eq (1)), is identical to this Boltzmann distribution at *T* = 1. This motivates the interpretation of *J*_*ij*_ as the “interaction” strength between viewers, although of course in practise the eye movements for different viewers do not interact (see Discussion). The algorithm for inferring *J*_*ij*_ from *C*_*ij*_ follows existing literature [21] and is described in S1, S2 and S3 Appendices.

### Modularity

Modularity is a useful measure of the existence and strength of communities in a network, with higher modularity meaning that more of a network’s links, and most of its strongest links, are between nodes that are members of the same community rather than between nodes that belong to different communities [29, 30]. The most conventional definition of modularity is based on a connectivity, or adjacency, matrix *A*_*ij*_ which has values 1 and 0 to indicate if two nodes are connected or not. We generate this adjacency matrix by applying a threshold to the interaction matrix: *A*_*ij*_ = *J*_*ij*_ > *J*_*o*_. Threshold *J*_*o*_ is selected for each video at a value where the largest cluster emerges following standard practice [34, 35]. To compute modularity each viewer has to be assigned to a cluster. Say the cluster number is given by *c*_*i*_. Modularity is then defined as:

$$\begin{array}{c} Q=\frac{1}{2a}\sum_{i}\sum_{j}\left[A_{ij}-\frac{a_{i}a_{j}}{2a}\right]\delta \left(c_{i},c_{j}\right), \end{array}$$ (10)

where *a*_*i*_ = ∑_*j*_ *A*_*ij*_, *a* = ∑_*i*_ *a*_*i*_, and Kronecker *δ*(*c*_*i*_, *c*_*j*_) indicates with 1/0 whether two viewers are in the same cluster or not. The best assignment of viewers to clusters is found by maximizing *Q*, for which efficient algorithms have been developed [31].

### Critical videos

As discussed above, temperature *T* captures the level of fluctuations of states ${\vec{\sigma }}_{i}\left(t\right)$ in time, and the maximum entropy distribution (Eq (1)) is the minimum probabilistic model which explains our system, equal to the Boltzmann distribution given by Eq (9) at *T* = 1. So, we set the “operating temperature” *T*_*o*_ of the system when the video is shown, equal to 1. At this point, we calculate the average of pairwise correlations *C*_*ij*_, or susceptibility *χ* of the system, and we then do analysis on the system at different temperatures *T* to see how the average energy of the system changes for different values of *T*. The rate of change is called the heat capacity, i.e. how much energy the system can absorb as the temperature *T* increases:

$$\begin{array}{c} C_{V}\left(T\right)=\frac{\partial {\left\langle E\left(\vec{\sigma }\right)\right\rangle }_{P_{T}}}{\partial T}\;, \end{array}$$ (11)

where the average now is over the Boltzmann distribution $P_{T}\left(\vec{\sigma }\right)$ at temperature *T* (Eq (9)). This average is computed using a Monte Carlo sampling technique (see S2 Appendix). The heat capacity can be expressed in terms of the variance of the energy [36]:

$$\begin{array}{c} C_{V}\left(T\right)=\frac{{\left\langle E{\left(\vec{\sigma }\right)}^{2}\right\rangle }_{P_{T}}-{\left\langle E\left(\vec{\sigma }\right)\right\rangle }_{P_{T}}^{2}}{T^{2}} \end{array}$$ (12)

**Fig 7:**
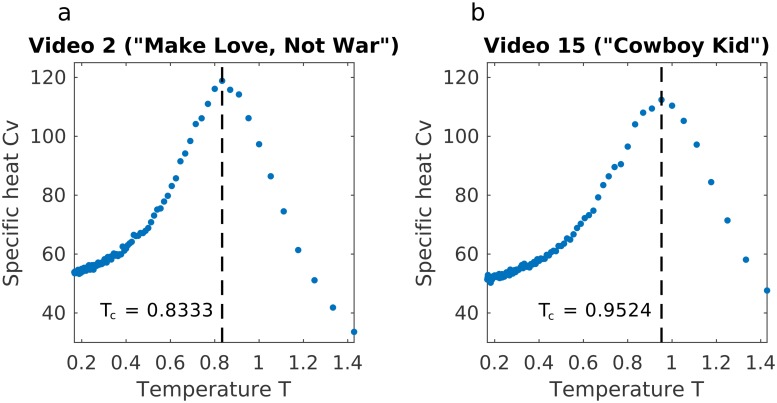
Critical temperature is found as the maximum of the heat capacity. *C*_*V*_ curves for the videos shown in (**A**) Fig 1A (the Axe Body Spray commercial “Make Love, Not War”) and (**B**) Fig 1B (the Doritos commercial “Cowboy Kid”), respectively; the vertical line indicates the peak in the curve of heat capacity, which gives the critical temperature *T*_*c*_ of the video. Since *T*_*c*_ for Video 15 (**B**) is closer to 1 (the operating temperature *T*_*o*_ of the video), this video is considered nearer to criticality.

A fundamental result from statistical mechanics is that the heat capacity is maximal—diverges in the thermodynamic limit—at a critical temperature *T*_*c*_. At this critical temperature a number of properties of the system are also maximal, including the susceptibility, or average correlation (Eq (7)) [36]. To find the critical temperature we therefore compute the heat capacity *C*_*V*_ (Eq (12)) by sampling from the Boltzmann distribution at different temperatures *T* and plotting the ratio of the operating temperature *T*_*o*_ to the temperature *T* [23]. We then follow the definition of the system being at a critical point when the heat capacity *C*_*v*_ diverges in the thermodynamic limit, or in the case of smaller systems such as our 25-subject group, reaches a maximum. We can determine the temperature at which the system reaches a critical point by plotting the heat capacity *C*_*v*_ of the system at temperatures *T* ranging from 0.17 to 1.43 and finding the temperature *T* at which there is a peak, which is the critical temperature *T*_*c*_ (Fig 7).

A video operating at its critical point when shown, such that the average correlation or susceptibility is maximal, would thus have a peak at *T* equal to 1. Values of *T*_*c*_ less than 1 imply a system in the liquid (random) phase (*T*_*c*_ < *T*_*o*_ = 1) and *T*_*c*_ greater than 1 would imply a system in the solid (ordered) phase (1 = *T*_*o*_ < *T*_*c*_). As the system becomes increasingly “liquid”—the temperature *T* at which *C*_*v*_ reaches a maximum, increases—the critical temperature tends toward zero, and as the system becomes more ordered, *T*_*c*_ approaches and then surpasses 1. In this study, we find that all of the videos have critical temperatures *T*_*c*_ less than 1, so no videos are operating exactly at criticality, but some reach criticality at *T*_*c*_ closer to the operating temperature 1 than do others. In keeping with theory, the videos with the operating temperature *T*_*o*_ closer to the critical temperature *T*_*c*_ display higher average correlations or susceptibility than those with a greater difference between *T*_*o*_ and *T*_*c*_ [36].

## Supporting Information

S1 Appendix The Maximum Entropy Method. (PDF)

S2 Appendix Monte Carlo algorithm. (PDF)

S3 Appendix Estimating *T*_*c*_. (PDF)

S4 Appendix Randomisation of trajectories. (PDF)

S5 Appendix Subject pool. (PDF)

S1 FigMonte Carlo values versus real values of *C*_*ij*_ and $\langle {\vec{\sigma }}_{i}\rangle$ for a sample video.Pairwise correlations (**A**) found experimentally are reproduced by the model found using the Monte Carlo learning algorithm (*r* = 0.99, *p* = 2.6 × 10^−273^, *N* = 300). Each point is a single pairwise correlation *C*_*ij*_. Average gaze direction (**B**) for each subject ${\vec{\sigma }}_{i}$ found experimentally are also reproduced by the model (*r* = 0.99, *p* = 9.4 × 10^−20^, *N* = 25 for x-component of direction, *r* = 0.99, *p* = 1.6 × 10^−19^, *N* = 25 for y-component of direction). For each colour, one point is one subject’s gaze direction. Red points denote the y-component and blue points denote the x-component. Black dashed lines have a slope of 1 to demonstrate the faithfulness of the recreated values.(EPS)

S2 Fig Specific heat curves for all videos. The operating temperature of each video is *T*_*o*_ = 1. Each video has a different critical temperature *T*_*c*_ defined at the peak of *C*_*V*_. All of the videos are near to criticality, but some videos are closer than others. These are also the videos with the highest inter-subject correlations and highest average couplings (see Results in the main text). The *C*_*v*_ curve of each video has a different style and colour of marker.
(EPS)

S3 Fig Maximum eigenvalues of the matrix of couplings *J*_*ij*_ correlate strongly with average couplings *J*_*avg*_ and critical temperatures *T*_*c*_ found from the Monte Carlo simulation. The maximum eigenvalue of *J*_*ij*_ (**A**) follows the same increasing trend with average coupling *J*_*avg*_ (*r* = 0.58, *p* = 9.9 × 10^−07^, *N* = 61) as does the critical temperature found from the Monte Carlo algorithm. (**B**) Although the critical temperature found by Monte Carlo simulation and the critical temperature estimated from the largest eigenvalue of the matrix of couplings *J*_*ij*_ are not equal, they are strongly correlated (*r* = 0.78, *p* = 1.2 × 10^−13^, *N* = 61). Each point represents one video.
(EPS)

S1 Table Key to S2 Table. (PDF)

S2 Table Subject demographics of our test group. (PDF)

S3 Table Identification of videos used. (PDF)

S4 Table Quantities related to the videos used. (PDF)

S1 Video Video of gaze trajectories, Doritos commercial “Cowboy Kid”. This video shows the gaze trajectories of the subject group for the duration of the video from Fig 1B of the main text (the Doritos commercial “Cowboy Kid”). There is a high level of synchronicity among the subjects’ gaze trajectories; moreover, several groups of subjects can be found, each group following a unique trajectory (as seen in the still shot in Fig 1b). In keeping with our results, this video is found to be close to a “critical point” when the correlations between gaze trajectories are modelled as interactions using the XY model.
(MOV)
