# Supplementary material for: Collective Behaviour in Video Viewing: A Thermodynamic Analysis of Gaze Position
Source: PLoS One. 2017 Jan 3;12(1):e0168995. doi: 10.1371/journal.pone.0168995 (PMC5207684; doi:10.1371/journal.pone.0168995)
Supplement: S1 Appendix — (PDF) [file pone.0168995.s001.pdf]

## S1 Appendix. The Maximum Entropy Method.

We seek a model for our data that will uncover any interactions between subjects which are not immediately apparent from the inter-subject correlations $C_{ij}$, and which will also disregard any "superficial" inter-subject correlations (i.e., two subjects seeming to correlate only because each has some similarities with the viewing pattern of a third subject, but not with each other). To find such a model for our data, we must solve an inverse problem; this is done using the Maximum Entropy Method. We seek a set of interactions that could reproduce the correlations $C_{ij}$ and average velocity of each viewer over all time $\langle\vec{\sigma}_i\rangle$ that were found from the experimental data—in terms of statistical mechanics, we know the expectation values of a system and now must find the coupling constants that reproduce these expectation values while maximising the entropy of the system. Our data was somewhat noisy even after it was cleaned, so it was especially important to find a model that would uncover the true interactions $J_{ij}$ and individual fields $\vec{h}_i$. Generally, when following the Maximum Entropy Method, a model will take the form of a probability distribution $P(\vec{\sigma}_i)$, as given in Eq (1) of the main text, and must produce the same values of correlations and means as found experimentally.

$$\begin{aligned} &\langle\vec{\sigma}_i\rangle_P = \langle\vec{\sigma}_i\rangle \\ &\langle\vec{\sigma}_i \cdot \vec{\sigma}_j\rangle_P - \langle\vec{\sigma}_i\rangle_P \cdot \langle\vec{\sigma}_j\rangle_P = C_{ij} \end{aligned} \tag{1}$$

Here, $\langle\cdot\rangle_P$ is the average taken over the distribution $P(\vec{\sigma}_i)$. In order not to make any assumptions beyond what has been measured experimentally, the only constraints on our model are those given in S1 Appendix Eq (1); this implies that any other information necessary to satisfy our model is randomised. Therefore, the entropy of the function $P$ must be maximised, giving the distribution found in Eq (1) of the main text.

This is the Gibbs distribution of a statistical mechanical model at temperature $T$ equal to 1 where $Z$ is the partition function, $J_{ij}$ are the couplings, and $\vec{h}_i$ are the fields. The particular model that best fits our problem is the fully-connected XY model, as the velocities have angles and unit length like the spins in the XY model. In this model, spins rotate in the plane, and in principle, each spin interacts with any other spin in the system. In order to have the theoretical values match the experimental values for correlations $C_{ij}$ and average velocities $\langle\vec{\sigma}_i\rangle$, $J_{ij}$ and $\vec{h}_i$ must be fixed. The theoretical quantities $\langle\vec{\sigma}_i\rangle^{th}$ and $C_{ij}^{th}$ are functions of $J$ and $\vec{h}$, as in S1 Appendix Eq (2). Moreover, since the distribution $P(\vec{\sigma}_i)$ is a Gibbs distribution at temperature $T = 1$, we can generalise to other temperatures as in Eq (9) of the main text.

$$\begin{aligned} \langle\vec{v}_i\rangle^{th}(J,\vec{h}) &= \langle\vec{v}_i\rangle \\ C_{ij}^{th}(J,\vec{h}) &= C_{ij} \end{aligned} \tag{2}$$
