# Supplementary material for: Collective Behaviour in Video Viewing: A Thermodynamic Analysis of Gaze Position
Source: PLoS One. 2017 Jan 3;12(1):e0168995. doi: 10.1371/journal.pone.0168995 (PMC5207684; doi:10.1371/journal.pone.0168995)
Supplement: S2 Appendix — (PDF) [file pone.0168995.s002.pdf]

S2 Appendix. Monte Carlo algorithm.

We find the correct values of J_{ij} and $\vec{h}_i$ by implementing a Monte Carlo learning algorithm which updates J_{ij} and $\vec{h}_i$ at each successive run τ of the simulation by the following rules:

$$\begin{aligned} \Delta J_{ij}(\tau+1) &= -\eta(\tau)[C_{ij}^{th}(\tau) - C_{ij}] + \alpha \Delta J_{ij}(\tau) \\ \Delta \vec{h}_i(\tau+1) &= -\eta(\tau)[\langle \vec{\sigma}_i \rangle^{th}(\tau) - \langle \vec{\sigma}_i \rangle] + \alpha \Delta \vec{h}_i(\tau) \end{aligned} \quad (1)$$

$\eta(\tau)$ is a time-dependent parameter and α is the damping; for our case we set $\eta(\tau)$ equal to $0.01(1 - \frac{\tau}{\tau_{max}})$ and α equal to 0.7. The maximum number of runs τ_{max} was 1000 but the Monte Carlo was terminated early if the maximum difference between any pair of C_{ij}^{th} and C_{ij} at run τ was less than 0.01.

We start by reading the gaze velocity data for each subject for a given video, normalising it, and then computing pairwise correlations C_{ij} and mean velocities $\langle \vec{\sigma}_i \rangle$. A matrix of couplings J_{ij} is initialised as well as a vector to store the individual fields $\vec{h}_i$; for our case initial values are set to 0.1 for both. A set of N unit vectors S (where N is the number of subjects) is generated with random directions. At each run, the system undergoes thermalisation for 200000 sweeps of the Monte Carlo and then takes measurements for an additional 200000 sweeps. At each sweep, there are N Monte Carlo steps taken; at each step, one of the angles S_i is changed by some random amount. If the change in energy of the system δE is negative, i.e. the energy of the system decreases, the change is kept. Otherwise, the change is made with probability $\exp(\frac{-\delta E}{k_b T})$ for temperature T of the system. For each sweep, the pairwise correlations and mean normalised velocities (gaze directions) are found following Eqs (5) and (6) of the main text, and then averaged over all sweeps at the end of the run to find C_{ij}^{th} and $\langle \vec{\sigma}_i \rangle^{th}$. The couplings and fields J_{ij} and $\vec{h}_i$ are then updated according to S2 Appendix Eq (1).

This is sufficient to infer the couplings and fields in our case, shown by the fact that the theoretical values match the experimental values of C_{ij} (S1 Fig A, $r = 0.99, p = 2.6 \times 10^{-273}, N = 300$) and the components of $\langle \vec{\sigma}_i \rangle$ (S1 Fig B, $r = 0.99, p = 9.4 \times 10^{-20}, N = 25$ for x-component of gaze direction, $r = 0.99, p = 1.6 \times 10^{-19}, N = 25$ for y-component of gaze direction) very closely. The theoretical values $\langle \vec{\sigma}_i \rangle^{th}$, however, are not quite as precise due to the extremely small magnitude of the average directions: since the average is taken over the entire duration of the video, $\langle \vec{\sigma}_i \rangle$ ends up being very close to 0. Parameters h_i are also all close to zero, which is expected as the mean eye movement direction is negligible (there is no net drift of gaze over the course of a video).

We can also calculate quantities such as the heat capacity C_v and magnetic susceptibility χ for each video. The critical temperature T_c is the temperature at which the heat capacity and magnetic susceptibility diverge. S2 Fig shows the specific heat curves gotten from the Monte Carlo; the critical temperature of each video is the maximum of its curve, while the operating temperature of all videos is set to $T_o = 1$ without loss of generality. It can be seen that all of the videos are close to a critical point (see Discussion section in the main text for details of what this means), however, those that are closest to a critical point also have the highest average inter-subject correlations and highest average couplings, meaning that the behaviour of the viewers is extremely cohesive.
