# Supplementary material for: Collective Behaviour in Video Viewing: A Thermodynamic Analysis of Gaze Position
Source: PLoS One. 2017 Jan 3;12(1):e0168995. doi: 10.1371/journal.pone.0168995 (PMC5207684; doi:10.1371/journal.pone.0168995)
Supplement: S3 Appendix — (PDF) [file pone.0168995.s003.pdf]

S3 Appendix. Estimating T_c.

Another method is to estimate the value of T_c using the maximum eigenvalue $\lambda_{i,max}$ of the matrix of couplings J_{ij} where the partition function Z of our system diverges in the thermodynamic limit, i.e., where there is a "phase transition". Self-couplings J_{ii} are equal to 0. Z is given by:

$$\int d\sigma e^{-\frac{1}{2}\sum_i \vec{\sigma}_i^2 + \frac{1}{2T}\sum_{i\neq j} J_{ij}\vec{\sigma}_i\vec{\sigma}_j} \tag{1}$$

and solved for a fully connected system (mean-field) to get:

$$Z = \prod_{i=1}^{N}(1 - \frac{\lambda_i}{T})^{-\frac{1}{2}} \tag{2}$$

The maximum eigenvalues of J_{ij} for each video follow a similar increasing trend with average couplings J_{avg} (S3 Fig A; $r = 0.58, p = 9.9 \times 10^{-07}, N = 61$) as do the critical temperatures found via Monte Carlo (see Fig 5 in the main text) though the correlation is somewhat weaker. The correlations of the average couplings J_{avg} with the maximum eigenvalue of J_{ij} are roughly the same as those with critical temperature T_c from the Monte Carlo algorithm. Although the maximum eigenvalues of J_{ij} do not match exactly the critical temperatures found from the specific heat curves yielded by the Monte Carlo algorithm, they are strongly correlated (S3 Fig; $r = 0.78, p = 1.2 \times 10^{-13}, N = 61$). For the model in our paper, which is fully connected and therefore solvable, this estimation is a useful way of obtaining relative critical temperatures of several finite systems without resorting to a Monte Carlo algorithm, which may be computationally costly for large groups because we use the XY model, which has continuous spins rather than just the binary of spin-up or spin-down.
