# Supplementary material for: Collective Behaviour in Video Viewing: A Thermodynamic Analysis of Gaze Position
Source: PLoS One. 2017 Jan 3;12(1):e0168995. doi: 10.1371/journal.pone.0168995 (PMC5207684; doi:10.1371/journal.pone.0168995)
Supplement: S4 Appendix — (PDF) [file pone.0168995.s004.pdf]

S4 Appendix. Randomisation of trajectories.

To check that our results cannot be found simply from random data, we shuffle the trajectories for each video across all subjects and all time so as to destroy any correlations. We then follow the same procedure as with our data taken from actual subjects: find the pairwise correlations and mean velocities; compute the couplings and fields; find the heat capacity at different temperatures T of the system using the Monte Carlo learning algorithm outlined previously; then calculate the modularity of the system. This is done for each video. As expected, we find low pairwise correlations due to the extent to which the data was shuffled, and low couplings. We also find that the critical temperature of each video approaches zero, meaning that the system is in a very random state. As before, the critical temperature T_c is really $T_c = \frac{T_o}{T}$, for temperature T when the specific heat of the system is at a maximum and operating temperature (temperature when the video is shown) $T_o = 1$. Then, for a critical temperature T_c approaching zero, the temperature T of the system when specific heat C_V is maximal must be very high, corresponding to a very “fluid-like” state.
