# Supplementary material for: Collective Behaviour in Video Viewing: A Thermodynamic Analysis of Gaze Position
Source: PLoS One. 2017 Jan 3;12(1):e0168995. doi: 10.1371/journal.pone.0168995 (PMC5207684; doi:10.1371/journal.pone.0168995)
Supplement: S5 Appendix — (PDF) [file pone.0168995.s005.pdf]

S5 Appendix. Subject pool.

For the 25-person subject pool, a wide range of demographics is represented in an attempt to accurately represent the variety of people living in the United States and being exposed to the advertising shown during the Super Bowl. S2 Table shows the breakdown of the subject pool over four categories: gender, age range, ethnicity, and whether or not they are a native English speaker.

For S2 Table, the demographic groups are coded as follows in S1 Table. A blank entry mean that the subject did not provide information for that category. There are more females than males in our sample (15 females, 10 males) and the mean age range is 18-25 years. The most prevalent ethnic group is Asian (12 reporting, out of 25 subjects) and over two-thirds of the subjects are fluent in English but not native speakers (16 reporting, out of 25 subjects).
