# Supplementary material for: Collective Behaviour in Video Viewing: A Thermodynamic Analysis of Gaze Position
Source: PLoS One. 2017 Jan 3;12(1):e0168995. doi: 10.1371/journal.pone.0168995 (PMC5207684; doi:10.1371/journal.pone.0168995)
Supplement: S1 Table — (PDF) [file pone.0168995.s009.pdf]

S1 Table. Key to S2 Table.

Gender	1 - male	2 - female			
Age range	1 - 18-25 years	2 - 26-30 years	3 - 31-35 years		
Ethnicity	1 - Caucasian	2 - African-American	3 - Latino	8 - Asian	5 - Multiple
Native speaker	0 - No (but fluent)	1 - Yes			
