# Supplementary material for: Collective Behaviour in Video Viewing: A Thermodynamic Analysis of Gaze Position
Source: PLoS One. 2017 Jan 3;12(1):e0168995. doi: 10.1371/journal.pone.0168995 (PMC5207684; doi:10.1371/journal.pone.0168995)
Supplement: S2 Table — (PDF) [file pone.0168995.s010.pdf]

S2 Table. Subject demographics of our test group. Our subjects represent a range of ethnic, gender, age, and language groups in order to better simulate the diversity of people viewing Super Bowl commercials across the United States. A blank entry means that the subject did not report this data.

Subject ID	Gender	Age range	Ethnicity	Native speaker
2	1	1	8	0
3	2	2	8	1
4	1	2	1	1
6	1	1	8	0
7	2	2	3	0
8	2	1	8	0
11	2	2	3	0
12	1	2	1	1
13	2	1	1	1
14	2	1	8	0
15	2	1	8	1
17	2	1	8	0
18	2	3	3	0
23	2	1	8	0
24	2	1	8	0
25	2	1	3	0
26	1	1	8	0
27	2	1	1	1
29	2	2	8	0
30	1	2	3	0
31	1			
32	2			
33	1	2	2	0
34	1			
35	1	2	8	0
