# Supplementary material for: Collective Behaviour in Video Viewing: A Thermodynamic Analysis of Gaze Position
Source: PLoS One. 2017 Jan 3;12(1):e0168995. doi: 10.1371/journal.pone.0168995 (PMC5207684; doi:10.1371/journal.pone.0168995)
Supplement: S3 Table — (PDF) [file pone.0168995.s011.pdf]

S3 Table. Identification of videos used. Identifying information for each of the videos used in this study is included below: the video number; the name of the commercial; and the product or company being advertised. (Continued on next page.)

Video Number	Name	Company
1	Doberhuahua	Audi
2	Make Love, Not War	Axe
3	No Room For Boring	Toyota
4	Equalizer	Bud Light
5	A Hero's Welcome	Budweiser
6	Puppy Love	Budweiser
7	Cup Therapy	Butterfinger
8	Gracie	Cheerios
9	Life	Chevrolet
10	Romance	Chevrolet
11	Ransacked	Chobani
12	Going All The Way	Coca-Cola
13	It's Beautiful	Coca-Cola
14	Cool Twist	Bud Light
15	Cowboy Kid	Doritos
16	The Spill	Dannon Oikos Greek Yoghurt
17	Campaign Film	H&M
19	Time Machine	Doritos
20	Trust Your Power	Duracell
21	The Right Music	Beats Music
22	Nearly Double	Ford
24	Bodybuilder	GoDaddy
25	Puppet Master	GoDaddy
26	Stratos	GoPro
27	Grandma's House	Time Warner Cable
28	Hum	Heinz
29	Hug Fest	Honda
30	Nice	Hyundai
31	Dad's Sixth Sense	Hyundai
32	Ian Is Up For Anything	Bud Light
33	Framily Plan	Sprint
34	GoldieBlox	Intuit
35	It's Crunch Time	Subway
36	Rendezvous	Jaguar
37	Restless	Jeep
38	Jerry Ricecake	NFL Network
39	Undomesticated Snowboarding	Labatt Blue
40	Long Live Dreams	American Family Insurance
41	Love Hurts	TurboTax
42	Empowering	Microsoft
43	Need for Speed	DreamWorks Studios
44	Negotiator Rises	Priceline

S3 Table. Identification of videos used. (Continued from previous page.)

Video Number	Name	Company
45	America's Import	Chrysler
46	Strike	Maserati
47	Get Hyped For Halftime	Pepsi Cola
48	There Since The First Halftime	Pepsi Cola
49	Slow Clap	CarMax
50	Offroading	smart USA
51	Face Off	Sonos
52	Sorry, Coke and Pepsi	SodaStream
53	A Better Web Awaits	Squarespace
54	The Amazing Spider-Man	Sony Pictures
55	The Phone Call	Radio Shack
56	The Truth	Kia
57	No Contract (Part 1)	T-Mobile
58	Transformers: Age of Extinction	Paramount Pictures
59	Invisible	Bank of America
60	Wings	Volkswagen
61	You Can't Do That	WeatherTech
62	We Killed The Long-Term Contract	T-Mobile
63	No Contract (Part 2)	T-Mobile
