# Supplementary material for: Collective Behaviour in Video Viewing: A Thermodynamic Analysis of Gaze Position
Source: PLoS One. 2017 Jan 3;12(1):e0168995. doi: 10.1371/journal.pone.0168995 (PMC5207684; doi:10.1371/journal.pone.0168995)
Supplement: S4 Table — (PDF) [file pone.0168995.s012.pdf]

S4 Table. Quantities related to the videos used. Pertinent quantities for each of the videos identified in S3 Table are included below: duration (in seconds); Ad Meter rating; average subject rating; average homophily O_{avg}; average pairwise correlation C_{avg}; average coupling J_{avg}; and critical temperature T_c. Blank Ad Meter data means no Ad Meter rating is available for a commercial. (Continued on next page.)

Video Number	Duration (s)	Ad Meter	Subject rating	O_{avg}	C_{avg}	J_{avg}	T_c
1	55.1	5.87	6.14	0.69	0.35	0.23	0.9091
2	25.4	4.92	6.40	0.74	0.29	0.21	0.8333
3	57.1	5.88	6.32	0.76	0.30	0.23	0.8333
4	27.0		5.03	0.71	0.27	0.22	0.8333
5	55.0	7.21	6.97	0.74	0.29	0.22	0.8000
6	55.0	8.29	7.54	0.77	0.37	0.25	0.9091
7	25.0	4.90	5.56	0.77	0.32	0.21	0.8696
8	25.0	6.75	7.36	0.77	0.33	0.24	0.8696
9	55.1	5.79	6.61	0.73	0.26	0.22	0.8013
10	55.1	6.18	6.73	0.80	0.30	0.22	0.8514
11	55.1	5.68	6.51	0.75	0.34	0.23	0.8893
12	55.1	6.42	5.97	0.73	0.36	0.25	0.9110
13	55.1	6.06	7.19	0.78	0.31	0.22	0.8712
14	27.8	3.89	4.08	0.70	0.23	0.22	0.7143
15	25.1	7.58	6.46	0.73	0.40	0.25	0.9524
16	25.0	6.15	6.83	0.77	0.37	0.25	0.9091
17	25.0	5.44	6.12	0.69	0.33	0.23	0.9091
19	25.0	7.13	7.60	0.76	0.34	0.24	0.8696
20	55.1		8.46	0.83	0.39	0.27	0.9307
21	57.0	5.44	6.27	0.74	0.29	0.21	0.8514
22	85.1	4.62	5.99	0.72	0.30	0.23	0.8637
24	25.2	4.04	5.13	0.68	0.31	0.21	0.8696
25	25.1	4.66	5.62	0.75	0.31	0.27	0.8333
26	25.0		6.15	0.71	0.30	0.21	0.8333
27	55.0		5.69	0.65	0.38	0.27	0.9307
28	25.0	6.05	6.64	0.78	0.35	0.24	0.9091
29	62.1	5.26	6.17	0.72	0.20	0.19	0.7294
30	26.1	6.10	5.77	0.77	0.33	0.22	0.8696
31	25.2	6.87	7.87	0.80	0.39	0.24	0.9524
32	56.5	5.60	6.39	0.66	0.33	0.23	0.8696
33	25.1	3.96	5.51	0.73	0.32	0.24	0.8333
34	25.0	5.49	5.60	0.72	0.31	0.22	0.8333
35	24.9	3.91	5.25	0.69	0.33	0.25	0.8696
36	55.0	5.78	7.61	0.80	0.36	0.24	0.8893
37	56.9	5.18	6.55	0.69	0.25	0.21	0.8000
38	25.5	5.19	4.02	0.69	0.31	0.24	0.8000
39	25.0		5.62	0.73	0.34	0.24	0.8696
40	55.0		6.21	0.72	0.26	0.22	0.8167
41	55.2	5.19	6.35	0.81	0.35	0.24	0.9348
42	55.1	6.65	8.31	0.82	0.33	0.24	0.8893

S4 Table. Quantities related to the videos used. (Continued from previous page.)

Video Number	Duration (s)	Ad Meter	Subject rating	O_{avg}	C_{avg}	J_{avg}	T_c
43	26.1	4.34	5.73	0.77	0.27	0.21	0.8333
44	26.0		6.02	0.74	0.29	0.21	0.8333
45	115.2	6.24	6.02	0.69	0.38	0.27	0.9300
46	85.2	4.85	6.46	0.76	0.26	0.22	0.8009
47	27.1	6.30	7.12	0.78	0.26	0.22	0.8000
48	25.0		6.63	0.79	0.31	0.22	0.8333
49	26.1	4.49	5.23	0.71	0.30	0.23	0.8696
50	27.6		6.85	0.83	0.33	0.25	0.8333
51	55.0	4.91	7.34	0.78	0.28	0.21	0.8167
52	27.1	4.66	5.70	0.70	0.28	0.20	0.8333
53	25.1	4.38	6.50	0.76	0.34	0.24	0.8696
54	28.1	4.93	7.12	0.73	0.22	0.19	0.7407
55	25.1	7.00	7.01	0.80	0.53	0.22	0.8696
56	57.0	6.09	6.35	0.74	0.28	0.22	0.8333
57	25.1	5.52	5.38	0.77	0.35	0.24	0.9091
58	28.2	4.61	6.67	0.69	0.27	0.21	0.8333
59	55.8	4.82	6.20	0.74	0.26	0.22	0.8167
60	55.0	6.24	7.48	0.78	0.33	0.24	0.8929
61	26.2	5.58	5.73	0.75	0.28	0.21	0.7962
62	25.1	4.77	5.76	0.71	0.33	0.26	0.8696
63	25.1	5.59	5.57	0.73	0.41	0.26	0.9524
